# Biological dosimetry for breast cancer radiotherapy: a comparison of external beam and intraoperative radiotherapy

**DOI:** 10.1186/2193-1801-3-329

**Published:** 2014-06-30

**Authors:** David K Woolf, Norman R Williams, Raheleh Bakshi, Seyed Yazdan Madani, David J Eaton, Sara Fawcitt, Katharine Pigott, Susan Short, Mohammed Keshtgar

**Affiliations:** Department of Academic Oncology, Royal Free Hospital, London, UK; Clinical Trials Group, University College London, London, UK; The Academic Breast Unit, University College London, London, UK; Leeds Institute of Cancer and Pathology, St James University Hospital, Leeds, UK

**Keywords:** Intraoperative radiotherapy, Breast cancer, H2AX, Cardiac toxicity

## Abstract

**Purpose:**

External beam radiotherapy (EBRT) is the gold standard adjuvant treatment after breast conserving surgery although a recent phase 3 trial has shown the non-inferiority of intraoperative radiotherapy (IORT).

Radiation exposure of the heart and cardiac vessels causes an increase in morbidity and mortality following EBRT for breast cancer.

We have used γ-H2AX foci formation in peripheral blood lymphocytes as a surrogate marker of dose delivered to the heart and great vessels and have assessed the feasibility of using this technique for biological dosimetry.

**Methods:**

34 patients were recruited, having either EBRT or IORT as part of a randomised controlled trial (TARGIT). Blood samples were taken prior to and after first fraction of radiotherapy, and the γ-H2AX biomarker then quantified.

**Results:**

Data were available for 31 patients. Following TARGIT-IORT there was an increase of 0.203 foci per cell (range −1.436 to 1.275) compared with 0.935 foci per cell (range −0.679 to 2.216) in the EBRT group; this difference was highly significant (p = 0.009). As TARGIT-IORT treatment is completed with a single fraction, whilst EBRT requires at least 15 fractions, the actual difference is estimated to be many times more.

**Conclusions:**

These data show a significantly greater change in γ-H2AX foci number per cell following one fraction of EBRT compared to TARGIT-IORT. This is the first study to demonstrate this effect using a biomarker and demonstrates a proof of concept methodology for similar applications.

## Introduction

Postoperative radiotherapy to the breast is regarded as an essential adjunct to breast cancer conservation surgery as there is overwhelming evidence that adjuvant radiotherapy decreases the risk of local recurrence and improves survival (Fisher et al. 1991; EBCTCG et al. 2011). However, whole breast radiotherapy is not without risk or side effects. Known adverse events include early skin erythema and desquamation as well as late skin fibrosis and telangiectasia, acute fatigue, late lung fibrosis, rib fractures, secondary malignancy and ischaemic heart disease (START Trialists’ Group et al. 2008).

Cardiac toxicity is the most likely of these to result in serious morbidity as well as mortality. A recent publication on the risk of ischaemic heart disease after radiotherapy for breast cancer (Darby et al. 2013) showed that the overall average of the mean doses to the whole heart was 4.9 Gy, and that the risk of a subsequent coronary event increased linearly with doses at a rate of 7.4% per Gy. It also concluded that the risk increase begins within a few years after exposure, and continues for at least 20 years. Data on whole body exposure has also shown an elevated risk of stroke and heart disease with doses over 0.5 Gy (Shimizu et al. 2010) and the association between breast radiotherapy and ischaemic heart disease is widely accepted (Sardaro et al. 2012).

Modern radiotherapy techniques are safer than those used in the past due to more accurate planning using computed tomography enabling an element of cardiac sparing, augmented by the existence of ‘heart atlases’ to avoid structures such as the left anterior descending coronary artery (Feng et al. 2011), an area at particular risk (Taylor et al. 2007). There are also data to show that using hypofractionated regimes result in a lower biological dose to the heart when compared with conventional schedules, due to the presumed relatively high fraction sensitivity of the heart (Appelt et al. 2013). Methods of reducing cardiac dose without compromising target coverage include shielding with multileaf collimation or breath-hold techniques (Bartlett et al. 2013a) but are not widely utilized (Bartlett et al. 2013b).

There has been growing interest in accelerated partial breast irradiation (APBI) allowing treatment to be delivered safely over a shorter duration. There are several APBI techniques available, which include linac-based intensity-modulated radiotherapy, multicatheter interstitial brachytherapy, balloon-based APBI, intra-operative radiotherapy using a mobile linear accelerator (ELIOT) or a miniature X-ray generator in the operating theatre (TARGIT) (Williams et al. 2011).

With the TARGIT technology, radiation is produced when accelerated electrons strike a gold target at the tip of a 10-cm-long drift tube with a diameter of 3 mm, resulting in the emission of low-energy X-rays (50 kV) in an isotropic dose distribution around the tip (Vaidya et al. 2001; Vaidya et al. 2002). The irradiated tissue is kept at a fixed, known distance from the source by spherical applicators to ensure a more uniform dose distribution. The tip of the electron drift tube sits precisely at the epicentre of a spherical plastic applicator, the size of which is chosen to fit the cavity after the breast cancer has been excised. Using this method, the walls of the tumour cavity are irradiated to a biologically effective dose (20 Gy to the tissue in contact with the applicator) that rapidly attenuates over a distance of a few centimeters and is likely to reduce the radiation exposure to non-breast tissue such as the cardiovascular system.

The early results of the TARGIT randomized trial indicates that the single dose of radiotherapy delivered at the time of surgery is safe and that for selected patients with early breast cancer it can be considered as an alternative to conventional whole breast radiotherapy (Vaidya et al. 2010). A five-year update of this trial has shown an increase in local recurrence in the TARGIT group [3.3% vs 1.3%], although it was considered to be non-inferior, with a non-significant trend towards improved overall survival with TARGIT [HR = 0.70 (0.46-1.07)] due to fewer non-breast cancer deaths [17 vs 35, HR 0.47 (0.26-0.84)]. There were 2 cardiac deaths in the TARGIT group vs 8 in the whole breast irradiation group (Vaidya et al. 2014).

Standard treatment planning systems allow the calculation of anticipated dose delivered by EBRT. A radiotherapy planning dosimetric study (Aziz et al. 2011) has shown that the low energy x-rays produced by IORT are likely to reduce the radiation exposure of the cardiovascular system compared with EBRT. However, there are currently no methods of obtaining physical dosimetry of the heart to ascertain the dose of radiation exposure actually received after radiotherapy delivery. This study aims to quantify the difference in exposure using a biomarker of radiation exposure: the phosphorylated histone H2AX protein (γ-H2AX). γ-H2AX is expressed after induction of DNA double strand breaks caused by ionising radiation (Valdiglesias et al. 2013), created as lymphocytes in the circulation pass through, and adjacent to, the irradiated field. This biomarker has been used for the assessment of the applied intergral body dose by radiotherapy to specific body sites (Sak et al. 2007; Sak and Stuschke 2010).

In this pilot study, we have used γ-H2AX foci formation in peripheral blood lymphocytes as a surrogate marker of radiation dose to the heart and great vessels with two aims: to estimate the doses of radiation actually delivered to the heart and great vessels with TARGIT-IORT and EBRT and to assess the feasibility of using this technique for biological dosimetry.

## Methods and materials

### Subject selection and treatment

Patients selected to enter this study had a diagnosis of early breast cancer suitable for breast conserving surgery and requiring radiotherapy. A diagnosis of Ductal Carcinoma in Situ (DCIS) was permitted in the EBRT cohort. The majority of patients entering this study were already enrolled in the TARGIT A study in which eligible patients were randomized between standard EBRT and IORT. Exclusion criteria included previous malignancies, bilateral breast cancer, any prior exposure to chemotherapy prior to study treatment, or exposure to radiation in the previous 28 days (excluding EBRT planning scans). Once treatments had been allocated either via the existing randomization structure of the TARGIT A trial or outside of the study using routine clinical practice, patients were approached to gain written informed consent for this study. Permission for this study was obtained from the local National Health Service Research and Ethics Committee.

TARGIT-IORT was given using low energy X-rays (50 KV), and a prescribed dose of 6 Gy at 1 cm from the applicator surface, following a previously described technique (Vaidya et al. 2010). This corresponds to approximately 20 Gy at the applicator surface. EBRT was given using standard 3-dimensional techniques using a dose of 40.05 Gray in 15 fractions (2.67 Gray per fraction) (START Trialists’ Group et al. 2008). Clinicians were free to use a boost to the tumor bed as required but this would have been given after the blood samples were taken so will not have affected the γ-H2AX analysis in this study.

### Laboratory

**Figure 1:**
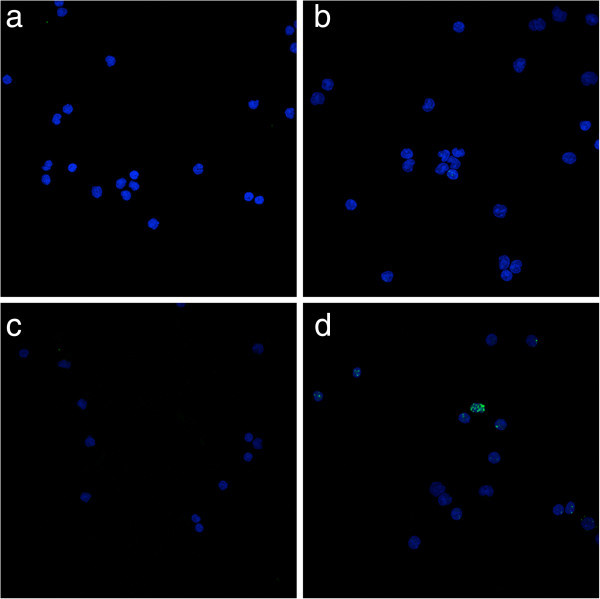
**Microscopy appearance of γ-H2AX foci (nuclei appear blue, foci appear green). a**: IORT pre RT, **b**: IORT post RT, **c**: EBRT pre RT, **d**: EBRT post RT.

Two 10 ml venous blood samples were obtained by the clinical team at the time of either first fraction of EBRT or at the time of TARGIT-IORT. The first sample was drawn 10 minutes prior to the radiation exposure to act as a baseline measurement and a second sample was taken 20 minutes after completion of the radiation exposure. Samples were rapidly transported to the on-site laboratory. Lymphocytes were isolated from 5 ml blood, with the standard continuous gradient separation using Isopaque-Ficoll technique. Isolated cells were placed on covered slide chambers (Labtech, Sussex, UK). The cells were fixed with 4% paraformaldehyde in phosphate-buffered saline (PBS) for 10 min, blocked with methanol and acetone respectively at 4°C, and then washed in 2 g bovine serum albumin (BSA) in 100 ml PBS (Sigma Aldrich, Dorset, UK). Primary antibody at dilutions of 1:300 (monoclonal, serine 139 phospho- H2AX; Millipore, Watford, UK) was then added and incubated at 4°C overnight. The slides were then washed with PBS/2 wt% BSA and incubated with secondary antibody (Alexofluor 488 Goat Anti-mouse Ig G at 1:400 dilution, Invitrogen, Paisley, UK) added for 1 h at room temperature. Slides were then washed in PBS/2 wt% BSA and mounted in 4′,6-diamidino-2- phenylindole. Slides were viewed with an inverted Leica SP2 confocal laser microscope for dual staining by sequentially scanning the two emission channels (488 and 514 nm). For foci counting, cells were viewed under ultraviolet illumination using a Nikon inverted micro- scope and 3100 objective (Figure 1). Foci were counted in at least 100 lymphocytes per slide, with three slides counted for at each sample (prior and after irradiation).

### Statistical analysis

Foci number per cell were calculated for each sample, and the within-patient change from baseline (pre-radiotherapy to 20 minutes post-radiotherapy) determined by subtraction. The mean differences of these changes between groups of women exposed to TARGIT-IORT and EBRT radiation were compared using a two-sided two-sample *t*-test. Summary statistics were calculated for the descriptive variables. Analyses were performed using SAS version 9.3 (SAS Institute, Cary, NC).

## Results

34 patients were recruited to this study of which 20 received EBRT and 14 received TARGIT-IORT. 3 patients (2 EBRT and 1 TARGIT-IORT) were not evaluable due to technical errors in the laboratory transport or processing meaning γ-H2AX foci results were unavailable. Data are therefore reported for 31 patients. Patient demographics and tumour characteristics are displayed in Table 1. In the TARGIT-IORT group the median size of the treatment applicator used was 40 mm (range 30 – 50), and the mean duration of treament was 28 minutes (range 21–45.8).

**Table 1:** **Patient demographics and tumour characteristics**

	n = 31
Mean Age	64.5 years (range 41.1-94.5)
Invasive ductal carcinoma	24 (77%)
Invasive lobular carcinoma	1 (3%)
Ductal carcinoma in situ (DCIS) only	6 (20%)
Median size of tumour	18 mm (range 0.2-53.5 mm)
	n = 25 (excluding DCIS)
ER +ve	23 (92%)
PR +ve	21 (84%)
HER 2 +ve	2 (8%)
Grade 1	10 (40%)
Grade 2	9 (36%)
Grade 3	6 (24%)

**Figure 2:**
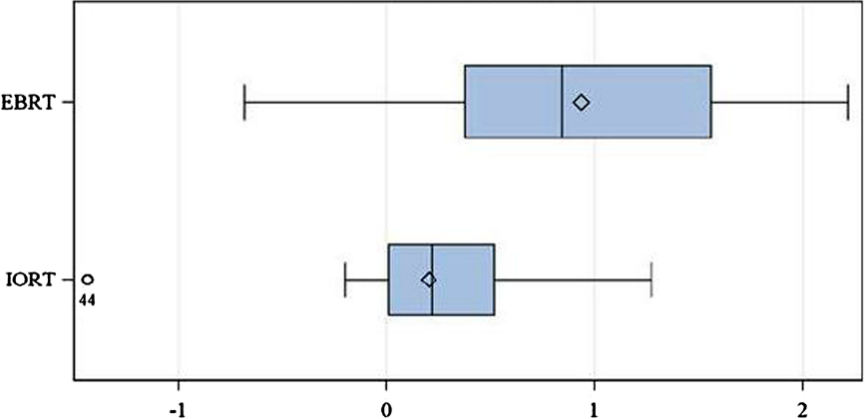
**Box-and-whisker diagram of change in foci number in each of the two treatment groups.**

Means and standard deviations for the change in γ-H2AX foci number per cell for each group are summarised in Table 2. Following TARGIT-IORT there was an increase of 0.203 foci per cell (range −1.436 to 1.275; 95% CI −0.180 to 0.586) compared with an increase of 0.935 foci per cell (range −0.679 to 2.216; 95% CI 0.556 to 1.315) in the EBRT group (Figure 2); this difference was highly significant by *t*-test (p = 0.009).

**Table 2:** **Change in γ-H2AX foci number per cell**

	IORT	EBRT
Mean	0.203	0.935
SD	0.633	0.764
n	13	18

With TARGIT-IORT, only a single fraction of radiotherapy is given, compared to 15 fractions of EBRT given as a course of treatment over 3 weeks. In order to compare the results of the course of radiotherapy, assuming no boost to the tumor bed and assuming equal changes with each fraction of EBRT (as the dose is constant), we have multiplied the EBRT data by 15. This gives a estimated change in total blood dose of γ-H2AX foci number per cell of 14.017 for EBRT compared with 0.203 for TARGIT-IORT which is highly significant (p < 0.0001).

## Discussion

In this study of patients receiving either standard external beam radiotherapy or intra-operative radiotherapy, a biomarker (mean number of γ-H2AX foci per peripheral blood lymphocyte) of DNA damage was found to be significantly higher in the EBRT group. It has previously been shown that there is a linear dose curve between radiation and γ-H2AX formation and that the steepness of the dose–response curve increased with the blood volume of the irradiated organ (Sak and Stuschke 2010). When these findings are applied to our data it suggests that the higher mean foci number are due to the larger irradiated blood volume within the EBRT field in comparison to the smaller TARGIT-IORT field. Part of the increase in irradiated blood volume will come from the vasculature in the breast tissue treated but it is likely that the majority of it will be due to radiotherapy dose to the heart and great vessels.

The clinical significance of these findings is of interest in a number of ways. The large difference between the increase in γ-H2AX foci number in the EBRT group and the TARGIT-IORT group, particularly after taking into account the 15 fractions of EBRT delivered, would suggest that the cardiovascular risk induced by TARGIT-IORT is minimal in comparison. This adds to its known safety profile in published data (Vaidya et al. 2010) and is in keeping with the known radiotherapy dosimetric profile of low dose (50 Kv) x-rays.

The large difference between the EBRT and TARGIT-IORT groups in terms of γ-H2AX foci number increase is likely to be due to a reduced total body exposure to radiotherapy in patients who received TARGIT-IORT, in addition to the reduced cardiac exposure. This is of particular importance in the rare but highly clinically important question of secondary malignancies. It is well known that high and low dose exposure to x-rays can induce cancers and previous work has addressed this question in patients receiving IORT, although using a phantom rather than patient specific data (Aziz et al. 2011). The reduction in whole-body radiation from treatment with TARGIT-IORT is likely to reduce the risk of a second cancer formation.

The use of biological dosimetry moves us forward from the current use of planning algorithms, which predict doses of radiotherapy received, towards a biological assay that shows radiation received and its effect on normal tissue in terms of double strand DNA breaks created. It also allows dosimetry in sites that are difficult to gain access to for physical dosimetric measurements, such as the heart. It would also provide a suitable method for a larger study to determine an association between radiation dose and the risks of heart disease and stroke (Darby et al. 2013; Shimizu et al. 2010; Haque et al. 2011).

The first expert consensus statement on screening for radiation-induced heart disease has recommended that patients who receive external beam radiotherapy for breast cancer should be screened for heart disease every 5 to 10 years (Lancellotti et al. 2013). This recommendation was made by the European Association of Cardiovascular Imaging (EACVI) of the European Society of Cardiology and the American Society of Echocardiography because of the increasing prevalence of radiation-induced heart disease due to the improved rate of cancer survival. The authors acknowledge that radiotherapy is now given in lower doses than in the past; nevertheless, there is still an appreciable risk of radiation-induced heart disease, especially when the heart is in the radiation field.

There are some limitations associated with this study. Although many patients with-in this study were part of the randomised TARGIT study, this study itself was not randomised which may have introduced an element of bias. DNA damage detected by this method will repair rapidly and as such are very sensitive to any delays from the administration of radiotherapy to the processing of the sample. We standardised this time in the study but some variation may still have occurred. This is especially the case as the length of time taken to deliver the TARGIT-IORT treatment is longer than EBRT so double strand repair might have occurred more in the TARGIT-IORT group. This study did not have statistical power to detect differences between treatments to the right and left side. Although we have hypothesised that the increase in γ-H2AX foci in the EBRT group is due to the increased volume of heart and great vessels in the field it is possible it is in fact from the increase in whole body dose which is difficult to account for. We hope to run a larger study in future.

It may be hypothesised that the reduced radiotherapy dose from TARGIT-IORT will reduce the risk of cardiovascular morbidity and mortality compared to EBRT. This is the first study to demonstrate the real time effect of radiotherapy to the heart and great vessels using a biomarker of biological dose and demonstrates a proof of concept methodology for similar applications.
